# The “ABCD” framework for assessment: a task-oriented approach to reconcile multimodal diagnostic discordance

**DOI:** 10.3389/fmed.2026.1885195

**Published:** 2026-07-08

**Authors:** Wubulikasimu Mijiti, Shadike Apaer, Xu Han, Qiang Dong, Xinling Cao

**Affiliations:** Department of Hepatic Laparoscopic Surgery, The First Affiliated Hospital of Xinjiang Medical University, Urumqi, Xinjiang, China

**Keywords:** artificial intelligence, esophagogastric junction, hiatal hernia, multimodal imaging, surgical decision-making

## Abstract

**Background:**

Pre-operative assessment of hiatal hernia (HH) often reveals discordance between endoscopy, barium swallow, and high-resolution manometry (HRM). These discrepancies can lead to diagnostic uncertainty and suboptimal surgical planning. We propose the “ABCD” framework to integrate these multimodal findings into a clinical-task-oriented decision process.

**Methods:**

A comprehensive review of current diagnostic standards (Chicago 4.0, Lyon 2.0, AFS-LDF classification) and emerging technologies, including artificial intelligence (AI), was conducted to identify common points of diagnostic discordance and their clinical implications.

**Results:**

The ABCD framework categorizes assessment into four essential domains: anatomical confirmation (presence and type), Barrier phenotyping (EGJ integrity using Hill and AFS-LDF systems), Clinical consequences (reflux burden and symptoms), and Decision support (integrating data for surgical planning). This framework clarifies that discordance—such as a large hernia on imaging with a functional barrier on HRM—often reflects different dimensions of the EGJ rather than test failure. AI-assisted endoscopic grading may further improve reproducibility, although its clinical utility requires external validation.

**Conclusion:**

The ABCD framework offers a structured, task-oriented approach for integrating multimodal HH assessment rather than a fully validated decision algorithm. By shifting focus from “the best test” to multidimensional clinical tasks, this framework may facilitate patient selection and perioperative decision-making, but its impact on clinical outcomes requires prospective validation.

## 1 Introduction

Hiatal hernia (HH) is not merely a common anatomical abnormality of the esophagogastric junction (EGJ) characterized by the displacement of abdominal contents into the mediastinum (1), but a complex biomechanical failure that disrupts the antireflux barrier. While its association with gastroesophageal reflux disease (GERD), Barrett's esophagus, and extra-esophageal symptoms is well-established (2, 3), its role in surgical planning remains a subject of intense debate. For surgeons, the presence of a giant or complex HH introduces critical risks, including gastric volvulus, obstructive ischemia, and iron-deficiency anemia (4, 5), which demand precise pre-operative mapping to optimize outcomes.

Despite its clinical stakes, the assessment of HH is plagued by diagnostic discordance. The EGJ is a dynamic environment, where axial displacement fluctuates under the influence of respiration, intra-abdominal pressure, and procedural factors (6, 7). The traditional 2-cm threshold for “sliding” hernias is increasingly viewed as a pragmatic compromise rather than a definitive biological marker, often leading to conflicting findings across different time points and modalities.

Current assessment relies on a multidisciplinary toolkit: barium swallow for anatomical contour, endoscopy for mucosal injury and valve morphology, and high-resolution manometry (HRM) for functional separation (2, 8). However, these tools often provide fragmented data, capturing isolated dimensions of a multifaceted disorder. Rather than viewing discordant results as “simple diagnostic error,” they should be recognized as complementary perspectives on the EGJ's integrity.

CT is useful for anatomical reconstruction in giant or complex HH, whereas pH or impedance-pH monitoring evaluates clinically relevant reflux burden. Therefore, discordance among these modalities may reflect different dimensions of the same disorder rather than simple diagnostic error.

Emerging standards, such as the American Foregut Society (AFS) LDF classification, represent a shift toward a more structured phenotyping of the EGJ barrier. In parallel, the integration of artificial intelligence (AI) offers a promising solution to mitigate the inter-observer variability inherent in endoscopic grading, although its clinical utility requires further validation (9–11).

In this review, we propose the ABCD framework (Anatomy, Barrier, Consequence, Decision support) to bridge the gap between fragmented diagnostic data and surgical action. By reframing HH assessment as a clinical-task-oriented process, this framework aligns multimodal findings with the specific questions essential for surgical decision-making.

## 2 Materials and methods

To ensure a comprehensive and clinically relevant evidence base for the proposed ABCD framework, we conducted a structured narrative literature search focusing on hiatal hernia assessment, esophagogastric junction phenotyping, and the clinical impact of diagnostic discordance.

We conducted a structured narrative literature search of PubMed/MEDLINE, Embase, Web of Science, and the Cochrane Library from database inception through May 6, 2026. The search strategy used a combination of Medical Subject Headings (MeSH) and free-text terms, including “hiatal hernia,” “esophagogastric junction,” “high-resolution manometry,” “barium swallow,” “Hill classification,” “American Foregut Society classification,” “AFS-LDF,” “pH-impedance monitoring,” “artificial intelligence,” and “machine learning.” To identify additional landmark studies, we also manually screened the reference lists of relevant guidelines, consensus statements, and key original investigations, including the Chicago Classification v4.0, Lyon consensus 2.0, the AFS-LDF classification white paper, and the Esophageal Diagnostic Advisory Panel consensus (6, 12–14).

Evidence selection was purposeful and clinically oriented. Priority was given to evidence-based guidelines, multi-society consensus statements, and prospective or large-scale observational studies that addressed HH diagnosis, EGJ phenotyping, diagnostic discordance, and pre-operative decision-making. Studies were synthesized narratively to identify modality-specific strengths, limitations, and clinically actionable points of disagreement across diagnostic tests. Because this article is a narrative review rather than a systematic review or meta-analysis, no formal quantitative synthesis, PRISMA flow diagram, or risk-of-bias assessment was performed. The primary objective was to integrate fragmented diagnostic concepts into a unified, task-oriented clinical framework. Given the substantial heterogeneity in HH definitions, diagnostic thresholds, test conditions, and clinically relevant endpoints across modalities, we did not attempt to establish a single diagnostic hierarchy or pooled accuracy estimate. Instead, the evidence was mapped to the four clinical tasks represented by the ABCD framework.

### 2.1 Diagnostic challenges in HH assessment

The persistent controversy surrounding HH assessment stems from the complex interplay between dynamic anatomy, modality-specific measurement biases, and divergent clinical priorities.

#### 2.1.1 Dynamic anatomy vs. static thresholds

EGJ is not a fixed anatomical landmark but a highly mobile functional zone. Its configuration is constantly modulated by respiration, deglutition, body position, intra-abdominal pressure, and even the technical nuances of the examination itself (8). This inherent mobility is most problematic in Type I sliding hernias. When the axial separation between the GEJ and the crural diaphragm fluctuates near the conventional diagnostic thresholds, minor variations in gastric insufflation or patient positioning can lead to binary “present vs. absent” reporting discrepancies.

Furthermore, the widely accepted 2-cm criterion should be recognized as a pragmatic diagnostic consensus rather than a rigid biological boundary (15). Small sliding hernias are better understood as part of a continuous spectrum of EGJ remodeling rather than a discrete “all-or-nothing” phenomenon. This perspective explains why a patient may present with borderline findings on endoscopy while showing overt herniation on a dynamic barium swallow or functional separation on HRM.

#### 2.1.2 Dimensional discordance: what are we measuring?

Diagnostic “disagreement” among modalities rarely indicates test failure; rather, it reflects the distinct dimensions of the EGJ captured by each modality (16):

- Endoscopy prioritizes luminal morphology and mucosal injury;
- Barium swallow elucidates dynamic transit and macro-anatomical configuration;
- High-resolution manometry (HRM) identifies the functional decoupling of the lower esophageal sphincter and the crural diaphragm;
- Computed tomography (CT) defines spatial relationships within the mediastinum;
- Reflux monitoring quantifies the physiological burden of barrier failure (17). Without a unifying framework, these fragmented data points can lead to clinical paralysis or suboptimal surgical planning.

#### 2.1.3 Task-dependent clinical endpoints

The definition of a “clinically significant” hernia varies by the surgical objective. In certain cases, the primary requirement is anatomical confirmation (type, size, and contents) to assess the risk of strangulation (13). In patients with refractory reflux, the focus shifts to whether the EGJ barrier defect correlates with objective acid exposure. Conversely, in giant or complex paraesophageal hernias, the evaluation must prioritize life-threatening complications such as volvulus, obstruction, Cameron lesions, or occult anemia (4, 5, 18). For those undergoing bariatric or anti-reflux surgery, the assessment must serve as a blueprint for procedural planning and risk stratification (19).

#### 2.1.4 Rationale for the ABCD framework

Therefore, the search for the “most accurate” test is fundamentally flawed unless the clinical task is first defined. A modality-centered approach misinterprets discordance as inconsistency, whereas a task-oriented approach leverages discordance to build a multidimensional phenotype. This logic provides the foundation for the ABCD framework, which organizes HH evaluation into Anatomical confirmation (A), EGJ Barrier phenotyping (B), Clinical consequence evaluation (C), and Decision support (D). Importantly, the ABCD framework is intended as an organizational and communication tool rather than a replacement for established diagnostic criteria or a validated treatment algorithm.

### 2.2 The ABCD framework for task-oriented HH assessment

**Figure 1:**
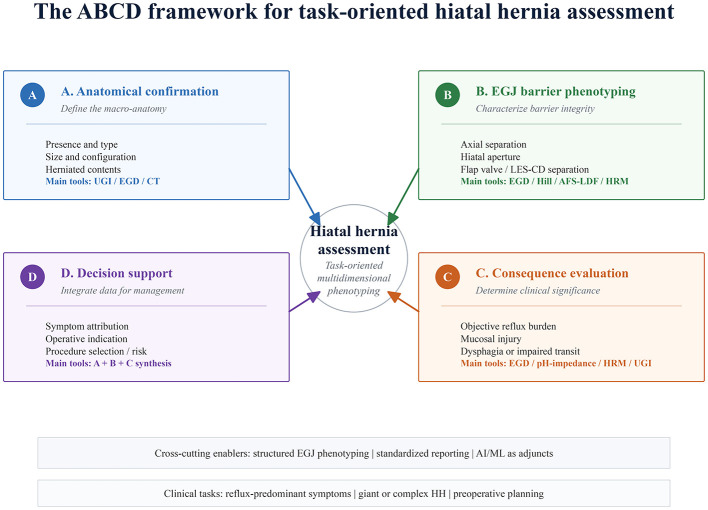
The ABCD framework for task-oriented hiatal hernia assessment.

The ABCD framework translates this task-oriented logic into four interdependent domains: anatomical confirmation, Barrier phenotyping, Clinical and functional consequence evaluation, and Decision support (Figure 1 and Table 1).

**Table 1:** The ABCD framework for task-oriented hiatal hernia assessment.

Domain	Primary clinical objective	Key questions addressed	Principal modalities	Main actionable output
A. Anatomical confirmation	Define the macro-anatomical blueprint of the hiatal defect	Is a hernia present? What is the type (I–IV), size, configuration, and herniated content?	Barium swallow/UGI series; endoscopy; CT	Anatomical diagnosis; surgical approach selection; mapping of complex or paraesophageal anatomy
B. EGJ barrier phenotyping	Assess structural and functional integrity of the anti-reflux barrier	Is the EGJ barrier impaired? What are the Hill grade, AFS-LDF features, hiatal aperture, and LES–crural diaphragm relationship?	Endoscopy with Hill and AFS-LDF assessment; HRM	EGJ phenotype; severity of barrier failure; distinction between anatomical and functional separation
C. Clinical and functional consequences	Determine whether the anatomical defect has objective pathological consequences	Is there objective reflux burden, mucosal injury, Barrett's esophagus, Cameron lesions, transit impairment, or motility disorder?	Ambulatory pH or impedance-pH monitoring; endoscopy; HRM; barium swallow/UGI series	Symptom attribution; objective evidence of GERD; complication and risk stratification
D. Decision support	Integrate multimodal findings for individualized management	Is intervention indicated? Which operative strategy is appropriate? What are the risks of dysphagia, recurrence, or complications?	Multimodal synthesis; clinical judgment; AI-assisted structured reporting as an investigational adjunct	Patient selection; procedure tailoring; perioperative risk mapping

#### 2.2.1 Domain A: anatomical confirmation (the surgical blueprint)

The primary task of Domain A is to establish the macro-anatomical “blueprint” of the defect. It defines the presence, type (I–IV), size, and configuration of the HH, as well as the nature of the herniated contents (1, 13). This domain is of paramount importance in patients with paraesophageal, giant, or recurrent hernias, where the degree of intrathoracic migration, gastric volvulus, or organ entrapment (e.g., colon or pancreas) directly dictates the urgency and complexity of the surgical repair (4, 5). Barium swallow and computed tomography (CT) serve as the cornerstone modalities here, providing the spatial orientation necessary for pre-operative anatomical mapping.

#### 2.2.2 Domain B: EGJ barrier phenotyping (structural and functional integrity)

Domain B shifts the focus from simple hernia size to the mechanical integrity of the anti-reflux barrier (ARB). Rather than treating HH as a binary finding, this domain evaluates the phenotypic architecture of the EGJ, including axial displacement, hiatal aperture diameter, gastroesophageal flap valve status, and the functional decoupling between the lower esophageal sphincter (LES) and the crural diaphragm. Standardized systems such as the Hill classification and the American Foregut Society (AFS) LDF classification provide essential structural phenotyping (12, 20), while high-resolution manometry (HRM) elucidates functional-anatomical separation between the lower esophageal sphincter and the crural diaphragm (21, 22). This domain is particularly critical for patients with borderline anatomical findings or reflux-predominant symptoms where the “quality” of the barrier is more relevant than the “quantity” of the hernia.

#### 2.2.3 Domain C: clinical and functional consequence (symptom attribution)

Domain C determines whether the anatomical defect has translated into objective pathological manifestations. This involves quantifying the reflux burden (via pH or impedance-pH monitoring), identifying mucosal injury (esophagitis, Barrett's esophagus, or Cameron lesions via endoscopy), and evaluating transit or motility impairments (via barium swallow and HRM) (2). This domain is essential for “symptom attribution”—distinguishing an incidental anatomical finding from a clinically significant disease that warrants intervention.

#### 2.2.4 Domain D: decision support (integrating data for action)

The final domain, Decision Support, synthesizes data from Domains A, B, and C to formulate a tailored management strategy. For clinicians involved in foregut disease management, particularly surgeons—this domain may support patient selection, selection of surgical technique (e.g., fundoplication type, mesh reinforcement, or gastropexy), and perioperative risk stratification (19). While artificial intelligence (AI) and machine learning offer promising avenues for automating EGJ grading and risk prediction, they currently serve as adjunctive tools to enhance diagnostic reproducibility rather than replacing clinical judgment (9, 23).

### 2.3 Reconciling discordance within the framework

Within the ABCD framework, discordance across modalities is interpreted as dimensional divergence rather than diagnostic failure. For example, a small sliding HH (Domain A) that exhibits severe barrier failure on HRM (Domain B) and pathological acid exposure (Domain C) may demand more aggressive anti-reflux intervention than its size would suggest. Conversely, a large paraesophageal hernia may require surgical repair based on its anatomical risk (Domain A) even if the reflux burden (Domain C) is minimal. By shifting from a modality-centered to a task-oriented interpretation, the ABCD framework provides a comprehensive phenotype that aligns with the multifaceted nature of HH.

### 2.4 Task-specific roles of conventional diagnostic modalities

The diagnostic armamentarium for HH evaluation comprises barium swallow (upper gastrointestinal series), endoscopy, high-resolution manometry (HRM), computed tomography (CT), and pH-impedance monitoring (24). While these modalities are frequently compared in terms of raw sensitivity and specificity, their true clinical utility is contingent upon the specific clinical task being addressed (25, 26). In the ABCD framework, these tools are not viewed as competing substitutes but as complementary instruments designed to capture different phenotypic dimensions (Table 2).

**Table 2:** Task-specific roles, strengths, and limitations of conventional diagnostic modalities.

Modality	Primary ABCD domain(s)	Main clinical role	Strengths	Limitations	Best-fit clinical scenarios
Barium swallow/upper gastrointestinal series	A, C, D	Dynamic anatomic mapping and transit assessment	Demonstrates hernia configuration, herniated contents, positional change, impaired transit, volvulus, and obstruction	Small sliding HH may reduce during the study; findings are influenced by swallowing, respiration, body position, intra-abdominal pressure, and protocol	Paraesophageal, mixed, giant, recurrent, or complex HH; intrathoracic stomach; suspected volvulus or obstruction; pre-operative anatomical mapping
Upper gastrointestinal endoscopy	A, B, C, D	Luminal inspection, mucosal assessment, and endoscopic barrier phenotyping	Identifies reflux esophagitis, Barrett's esophagus, Cameron lesions, strictures, erosions, ulcers, and gastroesophageal flap valve morphology; supports Hill and AFS-LDF assessment	Affected by insufflation, respiration, patient cooperation, image quality, and operator interpretation; limited assessment of complex extraluminal anatomy	Initial clinical evaluation; reflux-predominant symptoms; mucosal injury assessment; flap valve evaluation; pre-operative work-up
High-resolution manometry	B, C, D	Functional EGJ assessment and motility evaluation	Detects LES–crural diaphragm separation, double high-pressure zones, EGJ outflow obstruction, and major motility disorders	Does not define hernia sac size, herniated contents, volvulus, or complex thoracoabdominal anatomy; may be affected by catheter position and patient tolerance	Borderline HH; reflux-predominant symptoms; dysphagia; suspected motility disorder; assessment before antireflux surgery or HH repair
Computed tomography	A, D	Complex anatomical reconstruction and operative planning	Defines hernia extent, intrathoracic stomach, mediastinal relationships, involvement of other organs, post-operative anatomy, and complications	Static examination; not routinely required for small sliding HH; does not quantify reflux burden or EGJ barrier function	Giant, recurrent, post-operative, or anatomically complex HH; suspected intrathoracic stomach, volvulus, ischemia, or organ herniation
Ambulatory pH or impedance-pH monitoring	C, D	Reflux burden evaluation and symptom attribution	Quantifies acid exposure time, reflux episodes, non-acid reflux, and symptom association; provides objective evidence of GERD	Does not diagnose HH directly or define anatomy; interpretation depends on testing conditions and symptom recording	Reflux symptoms with absent, mild, or discordant endoscopic findings; pre-operative confirmation before antireflux intervention; persistent symptoms after repair

#### 2.4.1 Barium swallow: dynamic anatomic mapping (domain A and C)

Barium swallow remains the cornerstone for defining the macro-anatomical “blueprint” of the hiatal defect (27). Its primary strength lies in its dynamic capability to delineate the relationship between the EGJ and the diaphragmatic hiatus under physiological stress (e.g., swallowing and Valsalva maneuvers) (28). For surgeons, it is particularly useful for pre-operative mapping of paraesophageal, giant, or recurrent hernias, identifying gastric volvulus, and assessing transit impairment (29). However, the “snapshot” nature of the exam means that small sliding hernias may be missed due to transient reduction during the procedure, contributing to discordance with HRM or endoscopy (30).

#### 2.4.2 Endoscopy: integrating mucosal injury with structural phenotyping (domain B and C)

Upper gastrointestinal endoscopy serves a dual role: evaluating the luminal consequences of HH and providing a direct visual phenotype of the barrier (31). Beyond identifying esophagitis, Barrett's esophagus, and Cameron lesions (32, 33), the retroflexed view is essential for structural phenotyping using the Hill classification or the more granular AFS-LDF system (12). Despite its utility, endoscopic measurements are highly sensitive to gastric insufflation and patient cooperation, which can lead to over- or under-estimation of the axial hernia length (12, 28, 34, 35).

#### 2.4.3 High-resolution manometry (HRM): elucidating functional separation (domain B)

HRM provides a unique physiological perspective by identifying the functional decoupling between the lower esophageal sphincter (LES) and the crural diaphragm (CD) (21, 36, 37). According to the Chicago Classification v4.0, HRM is the superior tool for quantifying the EGJ contractile integral (EGJ-CI) and diagnosing occult motility disorders or EGJ outflow obstruction (38). While HRM excels at barrier phenotyping (Domain B), its inability to define the hernia sac volume or complex spatial anatomy limits its role as a stand-alone tool for large paraesophageal defects (25).

#### 2.4.4 Computed tomography (CT): spatial precision in complex defects (domain A and D)

While not routinely indicated for simple sliding hernias, multidetector CT is invaluable for “spatial mapping” in giant, recurrent, or anatomically distorted cases (29, 39). CT provides a static but high-resolution three-dimensional view of the mediastinal relationships and the involvement of other intra-abdominal organs. Its role is primarily in Domain D (Decision Support), where it aids in planning complex hiatal reconstructions and predicting the need for adjunctive procedures like gastropexy (40).

#### 2.4.5 pH and impedance monitoring: the proof of pathological consequence (domain C)

Reflux monitoring does not diagnose the anatomical presence of HH but quantifies its clinical impact (41, 42). This is the critical step in Domain C (Consequence), as it provides the objective “symptom-reflux correlation” necessary to justify surgical intervention (43). Monitoring is particularly crucial when anatomical findings are borderline or discordant, ensuring that HH repair is not performed for incidental findings that lack pathological significance (41).

### 2.5 Summary of modality integration

In summary, these modalities capture fragmented but essential dimensions of the EGJ: barium swallow maps dynamic anatomy, endoscopy assesses mucosal integrity, HRM phenotypes functional barrier failure, CT defines spatial complexity, and reflux monitoring quantifies pathological burden (8, 25). The clinical value of each test lies not in its ability to serve as a universal “gold standard,” but in its capacity to answer specific clinical questions within the ABCD framework.

### 2.6 Reconciling diagnostic discordance within the ABCD framework

Diagnostic discordance is a ubiquitous challenge in HH assessment (34). Traditionally, these discrepancies are attributed to suboptimal test sensitivity, inter-operator variability, or the inherent physiological mobility of the EGJ (1). While these factors remain critical—particularly in small sliding hernias where axial separation fluctuates—the ABCD framework suggests that discordance should not be dismissed as “diagnostic noise.”

Instead, discordance often yields vital phenotypic information. Each modality captures a distinct dimension of the EGJ's structural and functional integrity (44). For instance, a patient exhibiting “borderline” anatomy on barium swallow but demonstrating significant functional separation on high-resolution manometry (HRM) and pathological acid exposure (Domain C) may harbor a clinically relevant mechanical barrier failure, regardless of the hernia's axial size (45). Conversely, an isolated anatomical separation lacking objective reflux or mucosal injury should be interpreted as an incidental finding, warranting a more conservative management approach.

Within the ABCD framework, the clinical scenario dictates the resolution of discordance. In reflux-predominant cases, the focus is on symptom attribution via integrated phenotyping (43). In complex or giant hernias, the priority shifts to anatomical reconstruction, where structural imaging (Domain A) is paramount for operative planning and identifying complications like volvulus or incarceration (40).

### 2.7 Standardized EGJ barrier phenotyping

The clinical significance of an HH is not determined solely by its presence, but by the degree of anti-reflux barrier (ARB) impairment. Consequently, assessment has evolved from binary diagnosis toward standardized structural and functional phenotyping (44).

The Hill classification remains a foundational endoscopic tool, focusing on the morphology and competence of the gastroesophageal flap valve (GEFV) (20). However, the Hill grade is a localized assessment and does not independently quantify hiatal aperture or axial displacement (12, 31).

The American Foregut Society (AFS) LDF classification represents a significant advancement by decomposing EGJ integrity into three discrete surgical dimensions (12):

- L (Length): reflects axial displacement (Domain A).
- D (Diameter): reflects the hiatal aperture, which correlates with surgical complexity and crural repair tension.
- F (Flap Valve): reflects the structural integrity of the GEFV (Domain B).

This structured approach elucidates why hernias of identical axial length can manifest disparate clinical outcomes. A short hernia (low L) with a severely dilated hiatus (high D) and an incompetent valve (high F) signifies a profound mechanical failure requiring aggressive crural augmentation. By reframing HH as a multidimensional combination of structural features, the AFS-LDF system enables surgeons to move beyond simple length measurements toward a tailored procedural strategy.

### 2.8 AI-assisted assessment and machine learning

Artificial intelligence (AI) and machine learning (ML) offer a transformative potential for HH assessment by mitigating the subjectivity inherent in manual evaluation (46). For the gastrointestinal surgeon, the value of AI lies not in replacing clinical judgment, but in reducing inter-observer variability during the structural phenotyping of the EGJ—specifically regarding Hill grading and the AFS-LDF system (9, 11, 23, 46).

#### 2.8.1 Endoscopic recognition and automated grading

Current AI models focus primarily on the automated interpretation of endoscopic video and still images. These tools can assist in the real-time identification of HH and provide standardized grading of the gastroesophageal flap valve (10, 47). Such technology is particularly relevant for the “L” (Length) and “F” (Flap Valve) components of the AFS-LDF system, where consistent measurement is often confounded by gastric insufflation and endoscope positioning (10, 11). However, many existing models remain in the “proof-of-concept” phase, limited by single-center datasets and a reliance on static images that fail to capture the dynamic, respiratory-dependent nature of the EGJ (9, 23, 46).

#### 2.8.2 Multimodal data fusion for decision support

Beyond visual recognition, ML algorithms excel at integrating heterogeneous data streams—clinical symptoms, manometric metrics, and pH-monitoring results. Within the ABCD framework, this aligns with Domain D (Decision Support), where AI can assist in pre-operative risk stratification and predict post-operative outcomes such as dysphagia or recurrence risk (9, 23, 46). To achieve clinical maturity, AI-assisted assessment must move toward externally validated, real-time diagnostic adjuncts that provide interpretable, actionable outputs for the surgical team (Table 3).

**Table 3:** Potential applications and limitations of AI-assisted assessment in HH evaluation.

Application	Potential role in the ABCD framework	Main input data	Current limitations	Translational status
Endoscopic HH recognition	Domain A/B: identification and structured visual assessment	Endoscopic video or still images	Affected by insufflation, retroflexion angle, respiratory motion, and image quality	Early clinical validation
GEFV/Hill grading	Domain B: standardized flap valve assessment	Retroflexed endoscopic images	Training labels remain subjective; inter-center variability and annotation standards remain important barriers	Pilot validation
AFS-LDF reporting support	Domain B/D: structured reporting and surgical communication	Endoscopic video streams; structured L, D, and F annotations	Requires standardized acquisition protocols and validated annotation criteria	Investigational
Multimodal prediction	Domain D: risk stratification and decision support	Integrated symptoms, endoscopic findings, HRM metrics, reflux monitoring, imaging, and clinical variables	Heterogeneous inputs, limited external validation, and unclear interpretability	Investigational
Outcome prediction	Domain D: post-operative recurrence, dysphagia, or treatment-response prediction	Preoperative phenotype, operative details, and longitudinal surgical outcomes	Small datasets, variable endpoints, and limited long-term follow-up	Future adjunct

### 2.9 Scenario-based clinical pathways

**Figure 2:**
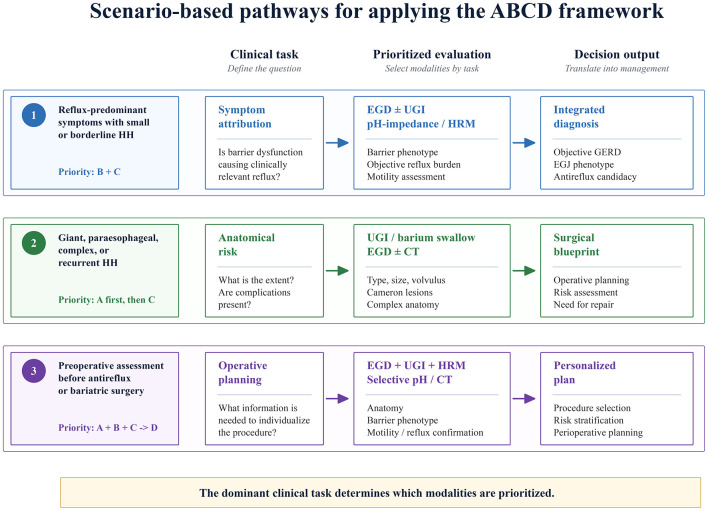
Scenario-based pathways for applying the ABCD framework.

The utility of the ABCD framework is best demonstrated when applied to specific clinical scenarios. Rather than a “one-size-fits-all” diagnostic battery, evaluation should be tailored to the patient's primary complaint and the expected surgical impact (Figure 2).

#### 2.9.1 Scenario 1: small or borderline HH with reflux-pre-dominant symptoms

In patients presenting with typical GERD symptoms and a small/borderline HH, the primary task is symptom attribution (Domain C). Endoscopy is the initial step to assess mucosal integrity and barrier morphology (13). When endoscopic findings are non-contributory or discordant, pH-impedance monitoring and HRM should be considered to confirm objective reflux burden, assess motility, and determine whether the anatomical defect is functionally linked to pathological reflux (19, 41, 42, 48).

#### 2.9.2 Scenario 2: giant or complex HH requiring anatomical risk mapping

For patients with paraesophageal (Types II–IV), giant, or recurrent hernias, the priority shifts to anatomical risk mapping (Domain A). Clinical evaluation focuses on identifying mechanical complications such as volvulus, incarceration, or Cameron lesions (49, 50). Barium swallow and CT with 3D reconstruction are indispensable here for surgical blueprinting and identifying herniated non-gastric organs, which fundamentally dictates the operative approach (25, 40, 51).

#### 2.9.3 Scenario 3: pre-operative assessment before antireflux or bariatric surgery

Preoperative evaluation for antireflux or bariatric surgery represents the most comprehensive application of the ABCD framework. The surgeon requires a complete synthesis of anatomy (Domain A), barrier integrity (Domain B), and functional consequences (Domain C) to personalize the procedure—such as deciding between a partial vs. total wrap, or the necessity of mesh reinforcement (14, 37, 52). In this scenario, the ABCD framework helps ensure that key dimensions of EGJ failure are considered during operative planning (40, 53). Artificial intelligence- or machine learning-assisted tools may eventually support this pathway by improving structured reporting or multimodal integration, but they should not replace conventional pre-operative evaluation.

These scenarios illustrate that HH assessment should be tailored to the dominant clinical task rather than applied as a uniform diagnostic battery.

### 2.10 Future perspectives and research priorities

Before a clinical-task-oriented approach to HH assessment can be universally implemented, several strategic priorities must be addressed:

#### 2.10.1 Harmonization of diagnostic taxonomies

The current reliance on arbitrary axial thresholds (e.g., 2 cm) fails to account for the dynamic and continuous remodeling of the EGJ. Future research must prioritize the development of consensus-driven reporting standards that integrate hernia length, hiatal aperture diameter, flap valve morphology, and functional separation assessed by HRM (12). Harmonizing these definitions across endoscopy, radiology, and manometry is the first step toward a unified “EGJ phenotype.”

#### 2.10.2 Linking phenotypes to surgical outcomes

Rather than comparing modalities based on diagnostic agreement alone, future studies should focus on prognostic validation. Future studies should include prospective, multicenter cohorts using standardized reporting templates for endoscopy, barium swallow, HRM, CT, and reflux monitoring. These studies should evaluate whether specific ABCD-domain combinations predict clinically meaningful endpoints, including objective reflux burden, symptom response, need for mesh reinforcement or gastropexy, post-operative dysphagia, radiological or symptomatic recurrence, reoperation, and quality of life. Transitioning from “test accuracy” to “outcome prediction” will allow the ABCD framework to serve as a true clinical-decision-support tool.

#### 2.10.3 Clinical-grade AI and multicenter validation

Standardized EGJ phenotyping provides the ideal foundation for artificial intelligence (AI)-assisted assessment. However, the field must move beyond technical feasibility toward prospective, multi-institutional validation. Future AI models should be evaluated by their ability to reduce inter-observer variability in real-time surgical workflows and their capacity to integrate multimodal data into actionable management plans (Domain D). Until such evidence is matured, the ABCD framework remains a pragmatic organizational model for synthesizing current best evidence.

### 2.11 Limitations

Several limitations should be acknowledged. First, this article is a structured narrative review rather than a systematic review or meta-analysis. Although we prioritized guidelines, consensus statements, and clinically relevant observational studies, no formal risk-of-bias assessment or quantitative synthesis was performed. Therefore, the proposed ABCD framework should be interpreted as a conceptual and task-oriented synthesis of current evidence rather than as an evidence-graded clinical guideline.

Second, the ABCD framework has not yet undergone prospective validation. Its domains are derived from existing diagnostic concepts and clinical reasoning, but the ability of specific ABCD-domain combinations to predict surgical complexity, post-operative symptom response, recurrence, dysphagia, or quality-of-life improvement remains to be tested in multicenter cohorts. Accordingly, the framework should not be used as a stand-alone treatment algorithm or as a substitute for established diagnostic criteria, multidisciplinary evaluation, or individualized surgical judgment.

Third, HH assessment is intrinsically heterogeneous. Differences in hernia definitions, axial length thresholds, patient position, gastric insufflation, respiratory phase, endoscopist or radiologist interpretation, HRM catheter position, and reflux-monitoring conditions may all influence test results. These factors are central to the diagnostic discordance addressed in this review, but they also limit the generalizability of any single integrated framework.

Finally, although AI-assisted assessment may improve reproducibility in EGJ phenotyping, most available models remain limited by single-center datasets, subjective training labels, variable image-acquisition protocols, limited external validation, and uncertain interpretability. At present, AI should be regarded as an adjunct for structured assessment rather than a replacement for clinical expertise.

## 3 Conclusions

The assessment of HH is fundamentally challenged by the dynamic nature of the GEJ and the inherent discordance among diagnostic modalities. Rather than pursuing a solitary “gold standard,” the ABCD framework reframes HH evaluation as a clinical-task-oriented process, integrating Anatomical confirmation, Barrier phenotyping, Clinical consequence evaluation, and Decision support.

By shifting the diagnostic paradigm from modality-centered comparison to multidimensional phenotyping, this framework aligns fragmented data with the specific questions essential for surgical planning. While prospective validation is required to quantify its impact on patient outcomes, the ABCD approach provides a structured and clinically practical framework for navigating the complexities of hiatal hernia assessment in foregut surgery.
